# A history of collective resilience and collective victimhood: Two sides of the same coin that explain Black Americans' present‐day responses to oppression

**DOI:** 10.1111/bjso.12562

**Published:** 2022-07-29

**Authors:** Hema Preya Selvanathan, Jolanda Jetten, Alexis Umeh

**Affiliations:** ^1^ School of Psychology The University of Queensland Brisbane Queensland Australia; ^2^ Boston University Boston Massachusetts USA

**Keywords:** collective continuity, collective memory, collective resilience, collective victimhood, social change, social representations of history

## Abstract

Collective victimhood and collective resilience are two sides of the same coin. However, most literature to date has focused on the experiences and consequences of collective victimhood. In the present research, we focused on the experiences of Black Americans, a group that has a legacy of victimization *and* resilience. As a part of Black Americans' collective memory, we explored the nature of historical collective resilience and examined its role in explaining collective responses to present‐day oppression, over and above any effect of historical collective victimhood. When they were asked to reflect on their group's history, across Studies 1 (*N* = 272) and 2 (*N* = 294), we found that Black Americans generated narratives of collective resilience. In both studies, we also found evidence that perceived historical collective resilience was linked to a greater sense of collective continuity, which, in turn, explained greater support for the ongoing Black Lives Matter movement. Our findings underscore the importance of considering narratives of resilience in a group's history and point to the way such collective resilience narratives can serve as a resource for the group in the present.

## INTRODUCTION

Out of the huts of history's shame, I rise.
Up from a past that's rooted in pain, I rise.
I'm a black ocean, leaping and wide,
Welling and swelling I bear in the tide.
Leaving behind nights of terror and fear, I rise.
Into a daybreak that's wondrously clear, I rise.
Bringing the gifts that my ancestors gave,
I am the dream and the hope of the slave.
– Maya Angelou.

As Maya Angelou recounts in her 1978 poem, groups that have a history of victimization often also have a history of resilience. The Black American community is a clear example of this. Throughout a long history of systemic oppression, Black people have also shown tremendous collective resilience in overcoming oppression. During the era of slavery in the 1800s, the Underground Railroad, which was a network of places such as churches, homes and barns, was developed to help free thousands of slaves from the Southern US Decades later during the Civil Rights era of the 1960s, thousands of Black Americans banded together to demand equal rights. Yet, social psychological literature to date has tended to overlook the ways in which a victimized group's history includes collective resilience. We propose that a victimized group's history of resilience may be intricately tied to the group's present‐day mobilization in response to ongoing oppression.

The goal of the current research is to better understand whether and how perceptions of historical collective resilience can provide a resource for group members in their fight for social change. We examined this in the context of Black Americans, and specifically, we asked two overarching questions: First, to what extent are narratives of historical collective resilience present in Black American's collective memory? Second, how does historical collective resilience explain present‐day mobilization against ongoing oppression? In addressing these questions, we simultaneously examined the role of historical collective victimhood because victimhood and resilience can be viewed as two interrelated constructs.

### Collective victimhood and collective resilience as two sides of the same coin

Being subjected to collective victimization—the infliction of harm by one group towards another—can continue to influence intergroup relations long after the atrocities have ended (for a review, see Noor et al., 2017) and collective victimization can affect group members who are not directly involved in the initial trauma (for reviews, see Bar‐Tal et al., 2009; Hirschberger, 2018; Noor et al., 2012). Collective victimhood refers to the subjective experience of collective victimization—including a sense of awareness of the victimization and the various ways in which individuals construe, interpret and make meaning of the collective harm and oppression that they experienced (Vollhardt, 2020). Although the literature thus far has focused almost exclusively on the negative or destructive dynamics and outcomes of collective victimhood, as we describe next, emerging work has noted that there is often a two‐sided nature to collective victimhood; specifically, there may also be collective resilience that emerges from experiences of victimization.

For one, although being a member of a victimized group exposes people to collective trauma, it can also promote psychological resilience by providing access to social support within the group (Muldoon et al., 2020). In the context of the conflict in Northern Ireland for example, victims were often recognized as resilient in coping with the trauma and stress of the conflict; thus the ‘victim’ label was associated with both weakness and strength (Ferguson et al., 2010). Further, research conducted in diaspora communities with a history or ongoing experiences of victimization found that participants freely discussed the juxtaposition between experiences of suffering, loss and struggle as well as experiences of survival, resilience and strength (Vollhardt & Nair, 2018; see also Jeong & Vollhardt, 2021 for an analysis of media discourse). Collective victimhood encompasses meaning‐making strategies that can result in negative (e.g. suffering, trauma) and positive outcomes (i.e. empowerment, strength). Taken together, these findings demonstrate that one of the ways members of victim groups make sense of their collective victimization is by developing narratives of resilience.

Further, related research on how groups make sense of their in‐group's history of victimization suggests that elements of resilience are often intertwined with victimization narratives. This has been found in the context of indigenous groups that have faced violence and displacement (e.g. Hatala et al., 2016; Ramirez & Hammack, 2014; Wexler, 2014). Amongst Aboriginal groups in Canada, historical representations of colonialism that solely emphasized victimization were viewed as stripping Indigenous people of their agency and therefore it was important to also highlight historical representations of resilience, especially to inspire other in‐group members (Neufeld & Schmitt, 2019). That is, representing the many ways in which in‐group members ‘fought back’ puts forth a strength‐based discourse that moves beyond viewing Indigenous groups as mere victims of colonialism (Neufeld & Schmitt, 2019). In North America and Australia, recognizing the continued survival of indigenous communities in response to collective trauma is central to building in‐group pride (Denham, 2008; Quayle & Sonn, 2019). Similarly, social representations of in‐group history from the perspective of Latin Americans highlighted not only the region's exploitation by European powers, but also the historical narrative of overcoming and fighting against these forces (Brasil & Cabecinhas, 2017).

Past research therefore suggests that historical narratives of collective resilience can develop alongside historical narratives of collective victimhood. Here it is worth noting that collective victimhood is distinct from historical collective victimhood—whereby collective victimhood encompasses the variety of ways that people make sense of their group's victimization (see Noor et al., 2017; Vollhardt, 2020), and historical collective victimhood more specifically refers to the shared trauma as a result of facing severe harm from other groups (Schori‐Eyal et al., 2014). In terms of how groups make sense of their history, we propose that historical collective resilience can be conceptualized as a distinct construct that is intricately positively related to historical collective victimhood.

#### Developing the notion of historical collective resilience

Although there is limited social psychological research specifically on the nature of historical collective resilience amongst oppressed groups, studying the psychology of resilience is not a new endeavour. Literature from clinical psychology has focused on understanding adaptive individual‐level responses to loss, trauma, or life‐threatening events. This work shows that victims demonstrate experiences of empowerment, agency and strength by responding to trauma through post‐traumatic growth, benefit‐finding and thriving (Bonanno, 2004; Linley & Joseph, 2005; Masten, 2007; Muldoon et al., 2019). Building on this work, collective resilience can be defined as a group's ability or capacity to achieve positive outcomes despite facing challenging or threatening circumstances that undermine a group's survival or vitality (see Bowleg et al., 2003; Sonn & Fisher, 1998). Related research has examined collective resilience in the context of mass tragedies such as natural disasters and terrorist attacks (Drury, 2012; Drury et al., 2009; Muldoon et al., 2020).

When it comes to collective resilience in response to structural discrimination and injustice, we argue that it is important for scholars to consider the history of resilience. One of the ways researchers have examined lived experiences of resilience is to study how people tell stories about their past. For example, a review of written autobiographical memoirs and narrated life stories by homosexual individuals in the United States found that they managed their stigmatized identity by constructing narratives of collective resilience (Hammack & Cohler, 2011). Similarly, despite facing stigma throughout their lives, it has been found that older LGBT adults aimed to leave behind a legacy of resilience for future generations (Bower et al., 2021). These autobiographical accounts tend to include clear instances of past experiences of resilience in the face of collective trauma. The stories of resilience typically revolved around positive sentiments of empowerment, strength and survival as a way of living with a marginalized group identity (Bower et al., 2021; Hammack & Cohler, 2011).

Building on this, we argue that it is important to specifically consider a group's history of resilience—that is, resilience specifically demonstrated by in‐group members in the past. For groups that have faced collective victimization, a key element of collective resilience is the resistance efforts undertaken by in‐group members in the past. Indeed, there is evidence that when marginalization and oppression has continued for generations, stories of in‐group resistance are transmitted within families from one generation to the next (Denham, 2008; Wexler, 2014). Further, Brown and Tylka (2011) found that Black Americans who have been socialized to appreciate their cultural legacy, such as by knowing their group's history of resistance, tended to cope better in response to discrimination. Similarly, many indigenous communities engage in storytelling of the ways in which their ancestors have survived oppression (Denham, 2008; Quayle & Sonn, 2019; Wexler, 2014). Therefore, just as there is intergenerational trauma, there can be intergenerational resistance (Atallah, 2017; Kazlauskas et al., 2017). When looking at documented accounts of Black history such as those portrayed in the US curriculum, there was evidence that the official textbooks captured narratives of resistance, for instance by including vivid depictions of how enslaved Africans revolted against institutionalized slavery (Brown & Brown, 2010). However, resistance was largely portrayed simplistically and reduced to the act of a handful of individuals during extraordinary moments, rather than a result of concerted, long‐term effort of the group (see also Salter & Adams, 2016).

Building on this past work, the present research aimed to examine whether and how a group's history of being resilient in response to oppression can serve as a resource for group members today. We define historical collective resilience as a perception tied to the in‐group's history, that is, the sense that a group has long been able to withstand, challenge and demonstrate strength in the face of oppression. There might be a sense of strength derived from having a group history of living through and fighting oppression, which can motivate present‐day group members to collectively cope with discrimination today. We expect that historical collective resilience would be an important part of a victimized group's collective memory, which will, in turn, explain present‐day responses to injustice, in particular through ongoing mobilization for social change. One way in which a group's past continues to influence a group's present is via a sense of enhanced collective continuity, that is, the perception that a group's past, present and future is interconnected. Thus, we further assessed whether perceived historical collective resilience predicts ongoing responses to injustice through a sense of collective continuity.

### Collective continuity as providing a link from historical collective resilience to present‐day mobilization

Collective continuity is the belief that the group's past, present and future are interconnected (for a review, see Smeekes & Verkuyten, 2015). It is characterized by viewing a continuation between the different historical events in a group's history, as well as viewing the group's cultural norms, values and practices as maintained throughout time (Sani et al., 2007). More generally, collective continuity is a sense that the group exists beyond the lives of individual group members and endures the vicissitudes of time (Sani et al., 2009), therefore, forming a basis for group identification (Sani et al., 2007). Since collective continuity is central to a group's functioning, people tend to be motivated to maintain a strong sense of collective continuity (Jetten & Hutchison, 2011; Jetten & Wohl, 2012; Smeekes & Verkuyten, 2014) and they may do so by drawing on particular historical narratives (Mols & Jetten, 2014; Obradović & Howarth, 2018).

Historical narratives are part of a group's collective memory, which refers to the ways in which groups remember their past, which has strong implications for a group's identity and sense of who they are (de Saint‐Laurent & Obradović, 2019; Hirst & Manier, 2008). Based on social representations theory, the shared values, ideas and practices within a group, including their history, allows individuals to make sense of the social environment and, therefore, create a shared understanding of one's social reality (Moscovici, 1998). Social representations of history are, therefore, intertwined with the broader political and social system within which an individual lives (Elcheroth et al., 2011). In the case of oppressed groups, we argue that they may specifically draw on their history of collective resilience to provide a sense of collective continuity in the context of responding to ongoing experiences of oppression.

In fact, it has been found that the collective memory of overcoming slavery provides a central a lens through which African Americans understand their collectively identity (Eyerman, 2004). When oppression is something that the group has long fought against, the actions and values of collective resilience of in‐group members from the past may come to be viewed as part of ‘who we have always been’ and ‘what we have always done’. Through perceptions of collective continuity, we propose that collective historical resilience can continue to provide reassurance, purpose and strength to transcend present‐day challenges (Hirschberger, 2018; de Saint‐Laurent & Obradović, 2019). Indeed, related research has shown that for groups with a history of trauma and suffering, preserving the in‐group's values and practices throughout time can help buffer against the negative outcomes of collective victimhood (Chandler & Lalonde, 1998; Wexler, 2014; see de Saint‐Laurent & Obradović, 2019 for a review). This is because history forms the basis of a group's identity by defining the in‐group norms, values and traditions (Liu & Hilton, 2005).

Different historical narratives can shape collective action in the present (for a review, see Freel & Bilali, 2021), including accounts that focus on collective victimhood and collective resilience. We propose that historical narratives that focus on collective historical resilience are particularly well suited to inspire resistance in the face of oppression today because such narratives promote a powerful connection between the past and the present (i.e. a source of collective continuity) as there is awareness of the group's longstanding tradition of fighting against and overcoming oppression.

While both collective victimhood and collective resilience in a group's history may theoretically contribute to greater perceptions of collective continuity, we reason that recognizing the in‐group's historical resilience is a particularly positive historical narrative that should contribute to in‐group pride and thereby enhance a positive group image. Even though collective victimhood may fuel perceptions of injustice and help spur collective action, if a group's history is remembered solely through the lens of collective victimhood (i.e. a kind of perpetual victimhood status, see Schori‐Eyal et al., 2017), it could also eventually lead to a sense of collective hopelessness that can be demotivating (see Aubin et al., 2016). Narratives of collective resilience on the other hand should provide various beneficial group‐based outcomes known to promote ongoing mobilization, such as a sense of group‐based efficacy and collective empowerment (i.e. ‘we did it before, so we can do it again’).

More generally, while to our knowledge no research to date has specifically investigated collective continuity as an antecedent to collective action, past work lends some support to the idea that perceiving an enduring connection with a group's history is an important tool in facing oppression in the present. For instance, the representations of Black history that highlighted the collective struggle of overcoming racism is linked to greater support for current anti‐racist policies because this helped people recognize the continued legacy of racism in the US (Salter & Adams, 2016). Similarly, a sentimental longing for the way society used to be in the past can drive collective action with the goal of re‐establishing collective continuity (Cheung et al., 2017). Further, thinking about the ingroup as including past, present and future generations increases willingness to endure suffering and make sacrifices in the interest of the group (Kahn et al., 2017). Theoretically then, a sense of collective continuity may be linked to present‐day collective action because it serves as a psychological resource for group members to feel that they are not alone in their collective experiences, thereby strengthening the resolve to defend the ingroup. Taken together, we propose that it is through a sense of collective continuity that historical collective resilience will help explain greater collective action outcomes in the present.

### Overview of present research

The present research focused on the context of Black Americans in the US—a group that has faced historical and ongoing oppression, as well as has a longstanding tradition of mobilizing for justice and freedom, which continues to this day. The goal of this research was two‐fold. First, through qualitative analysis, we investigated Black Americans' social representations of Black history in general (Study 1) and specifically in relation to the Civil Rights era (Study 2) to evaluate the extent to which narratives of historical collective resilience were present in their collective memory. We expected that in addition to narratives of collective victimhood, narratives of collective resilience will be a key part of the group's social representation of their history. Second, through quantitative analysis, we investigated whether historical collective resilience (versus victimhood) explained greater mobilization in response to ongoing oppression via a sense of collective continuity between the past, present and future of the group (Study 1 and 2). We expected that over and above any effect of collective victimhood, collective resilience would be associated with a greater sense of collective continuity, which would, in turn, predict greater support for the current Black Lives Matter movement. The survey material, data files and analysis scripts will be made available on the Open Science Framework (OSF) upon publication.

## STUDY 1

### Method

#### Participants

A sample of 303 participants with Black or African ancestry were recruited from the Cloud Research platform in August 2019 and were compensated with USD $1.00 for completing the survey. Sample size was determined based on cost and convenience. However, 31 participants did not answer the open‐ended survey prompt (e.g. copy‐pasted the question). Thus, 272 participants were retained for analyses. Participants' age ranged between 18 to 69 years (*M*_age_ = 36.76, *SD*_age_ = 11.07). There were 163 females, 107 males and 2 identified as non‐binary/third gender. Participants' median annual household income was between $40,000 to $59,999 with income brackets ranging between less than $19,999 to over $100,000.

#### Power analysis

A sensitivity analysis with G*Power (Faul et al., 2007) indicated that with a sample size of 272, we would have been able to detect a bivariate correlation of at least .17 with a two‐tailed α of .05 at 80% power, which matches the smallest correlation of interest that we found (*r* = .17 between historical collective resilience and support for the Black Lives Matter movement), suggesting that the study may have been adequately powered.

#### Procedure

Participants were asked to reflect on the history of Black people in the United States over the past century and then asked to write a few sentences (about 100 words) in response to the following prompt: ‘What would you say is an especially important moment in Black history and why was it important?’. Participants were asked to provide sufficient background information about the moment they chose, a description of the historical moment and to include vivid details by considering what it was like to be a Black person living in that era. To emphasize that we were interested in participants' own perceptions, the open‐ended writing box was given the heading: ‘Through my eyes: An important moment in Black history’. Participants also responded to a series of close‐ended measures on a scale ranged from 1 (*Strongly disagree*) to 7 (*Strongly agree*). The items within each measure were averaged to create composite scores. The order that participants completed the open‐ended and close‐ended measures was randomized (i.e. counterbalanced) to account for potential order effects.^1^

#### Measures

##### Historical collective victimhood

We used the items developed by Schori‐Eyal et al. (2014) which focused on the Israeli‐Palestinian conflict by adapting it to the context of Black people's history of victimization in the United States. Participants were asked the extent to which they agreed or disagreed with four statements regarding Black history in the United States (α = .74; i.e. ‘Black people have suffered unjust violence throughout history’, ‘Black people have long been victims of discrimination’, ‘The history of Black people is characterized by being victims of oppression’, ‘No matter what Black people do, some groups will always want to harm us’).

##### Historical collective resilience

We adapted the items used to measure historical collective victimization by changing the focus from victimization to resilience (α = .72; i.e. ‘Black people have fought for their rights throughout history’, ‘Black people have long been leaders of the struggle for equal rights’, ‘The history of Black people is characterized by resisting oppression’, ‘Although some groups will always want to want to harm us, Black people are resilient’).

##### Collective continuity

We adapted the 12 items on perceived collective continuity developed by Sani et al. (2007) to be about Black history (e.g. ‘Black people have preserved their traditions and customs throughout history’). Two items that were reverse‐scored had low item‐total correlation were, therefore, dropped from the measure. The remaining 10 items were averaged to create a composite score (α = .84).

##### Support for the black lives matter movement

We developed three items to assess attitudes towards the movement (e.g. ‘I support the Black Lives Matter protests’; α = .90).

##### Demographics

At the end of the survey, participants reported their age (in years), gender, approximate annual household income and political conservatism on a scale from 1 (*Very liberal*) to 7 (*Very conservative*).

### Results

#### Qualitative analysis: Reflection on Black history

##### Analytical approach

We applied qualitative content analysis (see Schreier, 2012) to analyse participants' open‐ended responses to the question on the most important moment in Black history, whereby the unit of analysis was each participants' written response to this prompt. Participants wrote an average of 132 words (*SD* = 56; Mdn = 115), ranging between 25 to 534 words. The modal number of words was 102 and 92% of participants wrote at least 100 words, which met the minimum number of words specified in the prompt. The goal of this analysis was two‐fold; first, to determine whether the concept of collective victimhood and collective resilience are reflected in participants' recollections of in‐group history (i.e. through categories), and second, to determine *how* collective victimhood and collective resilience are expressed (i.e. through subcategories).

In coding the data, we used a combination of inductive and deductive approaches. As a first step, the first author read all open‐ended responses and coded the data for ‘collective victimhood’ (i.e. instances of group‐based discrimination and suffering) and ‘collective resilience’ (i.e. instances of group‐based strength and overcoming adversity).^2^ These were broad coding frames derived from prior theorizing and, therefore, followed a deductive approach. All participants received a code based on whether they mentioned themes of victimhood and resilience (coded as 1 if a theme was mentioned and coded as 0 if a theme was not mentioned). Each theme was not mutually exclusive (e.g. participants may mention both themes or neither). The first author and a research assistant independently coded all responses based on this coding frame. There was a moderate level of agreement (see Altman, 1990; Fleiss & Cohen, 1973; Landis & Koch, 1977) between coders for both the victimhood code, κ = .55 (95% *CI*, .4618 to .6461), *p* < .001 and the resilience code, κ = .70 (95% *CI*, .5923 to .8074), *p* < .001. Differences were resolved through discussion.

Next, to identify subcategories within the theme of collective victimhood and resilience, we used an inductive approach. This was a secondary process to explain *how* each theme was expressed by participants, focusing on the type of events, experiences and topics that were discussed. This also aimed to enhance the validity of the themes. For this, the third author independently read all open‐ended data and developed initial categories to describe the content of each theme, along with relevant excerpts and reflections. Through repeated discussions between the first and third author, the categories were refined into subthemes that fell under each theme. Finally, the third author then checked if the subthemes (along with relevant excerpts) adequately captured each theme. Further modifications were made as needed. Thus, the process of coding the themes and subthemes were iterative. Note that when quoting participants' responses below, typing errors (e.g. misspellings, grammatical mistakes) were corrected.

#### Findings

Below we describe how themes of collective victimhood and resilience were expressed by participants.

##### Theme 1: Victimization

We found that 54.41% of participants (*n* = 148 out of 272) mentioned themes of victimization (see Table 1 for an overview of the subthemes and example quotes from participants). This theme focused on how Black people have collectively faced pain, suffering, loss and trauma throughout history. The first subtheme was institutionalized oppression, which referred to the systematic discrimination and unjust treatment of Black people that was legalized and backed by the state. Here, one major topic was the enslavement of African people in North America starting in 1619. After slavery was legally abolished, the era of racial segregation began (i.e. Jim Crow laws), which as one participant explained, ‘gave rise to racism and segregation to further prolong the suffering of African Americans’. Thus, when asked to describe Black history, participants described how legal doctrines, customs and practices throughout US history had systematically victimized Black people.

**TABLE 1:** Overview of collective historical victimization subthemes and example quotes from participants

Collective historical victimization subthemes	Example quotes
Institutionalized oppression	‘Throughout most of US history, Black citizens have suffered from extreme discrimination and racial harassment. They were forced to leave their lives in Africa and embark upon a journey to United States where they would be put to work as slaves.’
	‘An important moment in Black history is the year 1619, when slavery first came to the north Americas. This will serve as the pivotal moment in Black history that brings us to where we are today. The effects of the things that happened to our ancestors hundreds of years ago have never fully left us, nor have we been able to recover from this initial act of slavery. This has set the stage for all of the poor treatment that Black people have had to deal with and are still dealing with to this day.’
	‘…although Blacks were freed from slavery, they were not given equal access to what Whites had. The law at that point say things could be separate but equal, but it was not equal.’
Black death, murder and loss	‘The beating of Rodney King was a very dramatic event that unfold throughout the United States. That was the 1st time a video recording of a police officer beating a man senseless.’
	‘The assassination of Martin Luther King, Jr. was an important moment in Black history. He had become the chosen one to lead people through the Civil Rights era. […] He spent many days in jail for fighting the good fight. Unfortunately, on the fateful day, he was standing on a hotel balcony and he was shot. The movement was never the same. The people lost a leader that was never replaced.’
	‘I think a very important moment in Black history was the killing of Emmett Till in 1955. Emmett Till was a 14‐year‐old black boy who was visiting his family down south. Reportedly, Emmett and his friends went to a grocery store and had an altercation with a White woman. The White woman said that Emmett Till had made sexual remarks towards her. The woman told her husband. That night, the woman's husband and another man found Emmett Till and kidnapped him. […] The body was found and the men were charged. However, the men were found not guilty by a jury made up of other White people.’
	‘An important moment in Black history was the loss of “Black Wall street.” “Black Wall Street” was an area in Tulsa, Oklahoma where over a hundred thousand Blacks lived in affluent neighbourhoods, supporting Black‐owned businesses from restaurants to even hospitals in the early 1900's. Unfortunately, the “Tulsa Race Massacre” lead to its destruction as many racists burned homes, burned businesses, killed innocent Black men, women, children, all because of their race and ability to make a living without the assistance of their oppressors…’
Systematic exclusion of Black people from mainstream media representation	‘…there are a lot of historian figures in America that are Black and of African descent who were inventors. Most Black icons never get recognized for their work, for example Chuck Berry was the first Black person to create rock and roll; no one gives him credit, he is also ignored by mainstream music and society’.
	‘…us not knowing our real history keeps us in bondage to them. As long as we think we had no history before the slave ships, and that our most amazing moments were times we dared to defy the powerful “White Man”, they control the narrative’.

The second subtheme was on Black death, murder and loss, which referred to how racial discrimination led to the fatal victimization of Black people. One clear manifestation of this was the murders of unarmed Black people at the hands of law enforcement officials, as one as one participant described, ‘innocent Blacks have been killed by police and not many received any consequences for doing it’. Several high‐profile cases of police brutality were mentioned, including the murders of Rodney King by the Los Angeles Police Department in 1991 and Trayvon Martin by George Zimmerman in 2012. In addition, participants described the murders of important Black leaders of the 1960s Civil Rights movement including the assassinations of Dr. Martin Luther King Jr. and Malcom X. Participants also discussed the history of lynching and mob violence perpetrated by White Americans, in particular the brutal killing of Emmet Till, a 14‐year‐old African American after allegedly flirting with a White woman in Mississippi in 1955.

A third subtheme was the systematic exclusion of Black people from mainstream media representation. This refers to how the media has predominantly portrayed White Americans as the epitome of American society while at the same time excluding positive representation of Black people. One participant recalled: ‘I can remember back when I was a little girl and the album covers were not allowed to have Black people's faces on the album covers on them’ and another participant stated ‘you get stereotyped by the things you see on television’. Another explained how talent within the Black community was not acknowledged in broader American society, and important stories of Black history often get dismissed and swept under the rug. For example, one participant noted, ‘there are plenty of people that know nothing of Black peoples' struggle. It is sad that a lot of people want to act as if it didn't happen’. Taken together, participants' reflections demonstrated an element of subtle victimization that was defined through society's silence on Black people's accomplishments and history. This is in contrast to the more overt forms of victimization captured in the earlier two subthemes.

##### Theme 2: Resilience

We found that 81.25% of participants (*n* = 221 out of 272) mentioned themes of resilience (see Table 2 for an overview of the subthemes and example quotes from participants). This theme focused on Black people's collective strength and instances of overcoming adversity, including resistance to oppression. Part of this involved recounting collective struggles for liberation and justice in an empowering manner.

**TABLE 2:** Overview of collective historical resilience subthemes and example quotes from participants

Collective historical resilience subthemes	Example quotes
Black leadership in organizing collective action	‘A historical moment in black history for me is Rosa Parks refusing to get out of her seat on the bus full of White people for another White person. She stood her ground, and unfortunately was punished (wrongly in my eyes) by being arrested. She stood up for not only Black people but Black women and refused to back down because she knew even at that time that she had the right as anyone else of any colour to sit wherever she so chose. Back then it was a different, Whites (especially White men) tried to exercise so much power over Black people, even women. Rosa Parks wanted no part it this garbage and stood her ground for what was right.’
	‘Through my eyes an important moment in Black history occurred in November 1960. It happened with Ruby Bridges. In a small American stated a revelation for the Black movement. The whole of America watched as a brave 6‐year‐old Black girl walked into a segregated school. She was escorted and surrounded by federal marshals from the United States of America. She endured and bravely walked through a mob of screaming segregationists. She was a source of inspiration for us all. She walked into her school with the whole American people watching. This was an icon of the Civil Rights movement marked by Ruby Bridges.’
	‘An important moment in Black history was when Martin Luther King, Jr. took on leadership of the Civil Rights movement in the 1960's. Dr. King was a young Black minister raising a young family with his wife Coretta in Atlanta. Initially he was reluctant to take on the monumental task of becoming leader and spokesperson for this movement, probably because he excelled as a minister and wanted to be with his family, but once he took on the challenge, he infused the movement with passion, clarity, humanity and determination. Dr. King was an adherent to the passive resistance practiced by Ghandi, so participants in sit‐ins, marches, etc. were trained in this. King's was highly intelligent and his brilliant speeches were powerful, inspiring, motivating and awakened Blacks and Whites to the unjust situation faced by US Blacks, leading many from both races to come together to fight for Civil Rights. His contribution to the history and growth of the United States is massive, and has had positive repercussions around the world.’
Black autonomy and social independence	‘The reconstruction era is what I will say was a very important time for Blacks in the United States. During that time Black people learned to read, the largest group in the USA to move from illiteracy to literacy in such a short period of time. Black people continued to create and invent. Black cities were built, and Black people were able to have their own communities. Being from the South here in the United States, there are many landmarks that speak to who Black people were able to establish banks, care for land, build homes. All these things led to the wonder of the early 1900s.’
	‘During the rise of the Black Panther party, Black people were consciously waking up and unapologetic about loving themselves in their natural state. They encouraged growth, learning, wisdom and placed economic growth in their own hands. They also realized that it was on United States to strengthen and serve our communities, “we the people” had to strengthen our own people, instead of waiting for the government to do it. […] they were feeding children in their neighbourhoods, delivering food to low‐income houses, teaching Black people about their history and constitutional rights, helping people go through college, protecting their own, and showed a level of structure and organization of protest and change, that is strongly needed within the Black community. It was a Black Renaissance on a different level…’
	‘The Harlem Renaissance was a very important moment in Black history, because Black people proved to the world that we are talented, creative, artistic and intelligent. Artistic and intellectual accomplishments through the Harlem Renaissance were plentiful, and they were positive reflections of the capabilities and potential of Black people. […] The Harlem Renaissance proved that we did not need acceptance or approval from Whites to demonstrate our greatness. That is the true solution to the Black “struggle”…’
Trailblazers	‘An important moment in Black is when we inaugurated the first African American president. It was a pivotal moment for us and gave us so much hope for the United States. I was in fifth grade and I remember every Black person in my class feeling so proud and we were all almost in tears because that's the highest title you can have in the United States and for a Black person to have it made all of us so happy. To me this was such an important moment in history and every young Black person really looked up to him and knew we could do anything.’
	‘…Some of the historically most important Black people are those who are the trailblazers of being the first Black person in their field or specialty. As my example, I'd like to mention about two black astronauts, Guion Bluford and Mae Jemison. I'm personally more familiar with Mae Jemison, but I've heard of Bluford as well. Since these two have proven that Black people can become astronauts, one of the most difficult careers to advance, I'm convinced that any Black person can follow the path they set. […] The most important thing for the Black community going into the future is that they feel they can become anything they set their mind to.’
Legislative changes	‘The landmark case of Loving v, Virginia in 1967 is a case that gave a legal right for couples of different ethnicities or races to marry. This case came at a time when Civil Rights were being tested and defended to the greatest extent since the US Civil War. […] This case specifically involved Mildred Loving (a Black woman) and her husband Richard (a White man) who were sentenced to jail for marrying each other. They were violating the Racial Integrity Act of Virginia. After the Lovings won their case, Virginia's anti‐miscegenation law was outlawed. This is an important moment to me because it legally allows Black people to marry outside their race if they want to…’
	‘…The single most important indicator of shifting tides in the United States was the passage of the Civil Rights Act in 1964. This legislation barred discrimination on the basis of race, gender, religion, or national origin. More specifically, this act led to the desegregation of schools and public facilities as well as the protection of the right of African Americans to vote…’
	‘I consider Juneteenth to be a particularly important moment in Black history. After the Civil War, there were slaves throughout the country, especially in the deep South, who were unaware that the war had ended, the North had won, and that slaves were to be freed per Abraham Lincoln's Emancipation Proclamation. As slave owners controlled the information that slaves knew, it was possible to maintain this lie for an extended amount of time. Juneteenth marks the day that the last slaves in the South were told that, essentially, they were free. It is a celebration of the last formal vestiges of slavery coming to an end. For Black Americans, it is an acknowledgement of the vast systems that White people will uphold in order to continue exploitation of Black people, and the resilience that Black people have to continually oppose and free themselves from such systems.’

The first subtheme speaks to Black leadership in organizing collective action. Many participants described key protests events that Black people led during the 1960s Civil Rights movement. Participants recalled the efforts of prominent Black leaders including Harriet Tubman, Rosa Parks, Dr. Martin Luther King Jr. and Malcom X. For example, they referred to the significance of Dr. King's efforts, including the mass protests he organized and the memorable speeches he delivered (e.g. the ‘I Have a Dream’ speech in Washington D.C.). Several also described how Rosa Parks' actions sparked the Civil Rights movement, and there was a sense of gratitude for the sacrifice of Black leaders who paved the way for the changes observed in that era. In addition, participants referred to the various protest actions that occurred during the Civil Rights movement to desegregate the South, such as the Montgomery bus boycotts and the young Black students (known as the ‘Little Rock Nine’) who desegregated a high school at Little Rock, Arkansas.

The second subtheme was on Black autonomy and social independence, which refers to how Black people were thriving culturally, socially and economically. This was largely during the Reconstruction Era in the 1860s‐70s after enslaved Black people were freed. Examples include the Black Wall Street in Tulsa, Oklahoma and Durham, North Carolina, which were areas that were established by previously enslaved Blacks in the 1900s. Prosperous Black businesses, financial amenities and community services were set up independently from White‐run institutions to serve Black communities. Another example was the role of the Black Panther Party in promoting self‐determination amongst Black people and cultivating pride in Black culture. In addition, the thriving of Black culture was also evident in the Harlem Renaissance during the 1920s, primarily centred in Harlem, New York City—which was a time when African American culture boomed in music, art, fashion, scholarship and literature. Relatedly, another participant talked about the birth of hip‐hop music in the Bronx, New York City in the mid‐1970s as an important contribution from and for the Black community. Thus, the celebration of Black culture and achievements were viewed as an act of collective resilience against societal structures and institutions that were largely created by and for White communities.

A third subtheme is on the trailblazers in areas of politics, art, music, fashion and sport. By far the most mentioned figure was Barack Obama as the first person of African descent to become a US President. One participant discussed how President Obama's inauguration uplifted the Black community, both as a symbol of change and as a role model for others, stating ‘it felt like a weight that was holding our community down had been lifted’. Many participants detailed a sense of collective pride and excitement around Obama's inauguration. Beyond the political sphere, participants also mentioned representation in the beauty, music and sports industry, such as Jay‐Z as a famous Black musician who has grossed over a billion dollars and Jackie Robinson as the first African American to play in the Major League Baseball. Taken together, these Black icons were viewed as breaking barriers because their accomplishments were viewed as a collective win for the Black community in the broader fight against structural and societal racism.

A fourth subtheme that participants discussed was the notable legislative changes achieved that were signs of progress and were celebrated as such. This included the 13th Amendment to the US Constitution, which legally abolished slavery, the 14th Amendment, which granted citizenship persons born or naturalized in the United States including former slaves, and the Civil Rights Act of 1964. The commemoration of Juneteeth (19th June), which is a Black holiday that honours the emancipation of African Americans who were enslaved, was also mentioned. These legal victories were at times cautioned with the recognition that they did not completely resolve Black victimhood. The legal changes were, therefore, viewed as symbolic at times because the struggles were being legitimized and partly addressed within the legal system, yet those laws were often not immediately enforced, faced intense backlash from White communities and served to prolonged Black victimization.

#### Interconnections between resilience and victimization

It is important to note that the theme of resilience was often described in response to themes of victimization. Specifically, 66.22% of participants who mentioned themes of victimhood also mentioned themes of resilience (*n* = 98 out of 124 participants). These responses would start out by detailing the victimization that Black people have faced and then end by describing the resistance efforts led by Black people. This was especially the case for the subthemes on the Civil Rights movement. To explain why protest actions were organized, it was necessary to point to the victimization, which led to it. For example, this participant's response captured the back‐and‐forth between victimization and resilience throughout Black history:

The history of African Americans begins with slavery… The fate of slaves in the United States would divide the nation during the Civil War… after the war, the racist legacy of slavery would persist, spurring movements of resistance… Through it all, Black leaders, artists and writers would emerge and help shape the character and identity of a nation.

Similarly, in response to various tragedies, participants pointed to how they inspired mass mobilization for social change. In particular, the lynching of Emmet Till, a young Black boy who was accused of offending a White woman, was described as the moment that ‘sparked the Civil Rights movement’. Episodes of brutal victimization was viewed as a starting point of collective resilience. As one participant explained the significance of having an open‐casket funeral for Emmett: ‘…the world was able to see what was going on in the South. This got the people angry and rallied the Americans to their side…’ To take another example, the police brutality that occurred in response to the march in Selma sparked greater mobilization for change. One participant described how the government's attempts to repress the movement helped raise public sympathy:

The response to those marchers demonstrates how Americans have always had a history of perpetrating violence against the marginalized and their allies when the marchers were attacked… [The march] was televised, thus holding an ugly mirror to the American public about the evils and complacency that they have allowed to remain… it became more difficult for the general public and elected officials to brush the Civil Rights movement underneath the rug….

Beyond collective action, victimization also led to a sense of survival and coping as part of resilience. There is strength and bravery in facing oppression, and the mere continued existence of Black people was a source of collective pride. This was captured in the following participant's response in detailing Black people surviving slavery:

… We had to be strong just to survive those conditions. […] Foreign lands were our new homes. Trying to teach your children to be self‐appreciative and to survive becomes your focus, your will to live. Fast forward to today, our ancestors taught us survival and perseverance. We endured the worst but achieved the most….

As these responses illustrate, participants explicitly mentioned resilience as a response to victimization and is, therefore, consistent with our view that victimization and resilience may be two sides of the same coin.

#### Quantitative analysis: The link between historical collective victimhood and present‐day responses to injustice

**FIGURE 1:**
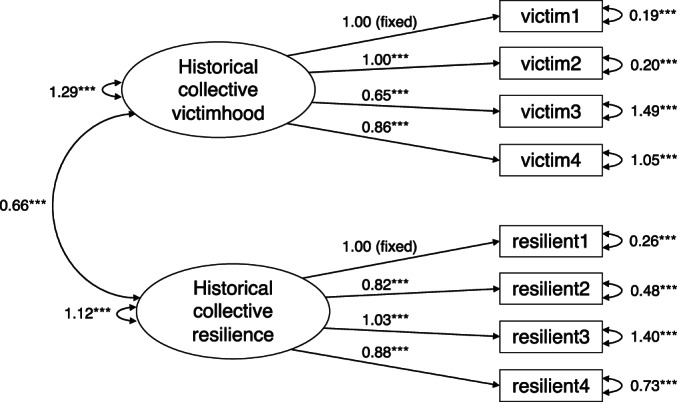
Two‐factor confirmatory factor analysis of historical collective victimhood and historical collective resilience for Study 1

Corroborating the qualitative analysis, our quantitative analysis first focused on testing whether the collective victimhood and resilience beliefs items represented two distinct constructs. To do so, we conducted a confirmatory factor analysis (CFA) using the CALIS procedure in SAS 9.4. A two‐factor solution was specified whereby (1) the four collective victim beliefs items loaded onto the first latent factor and (2) the four collective resilience beliefs items loaded onto the second latent factor. We fixed the loading of one of the indicators for each factor to a constant (i.e., 1) (see Kline, 2015). The model fit was acceptable, *χ* ^2^ (17) = 45.21, *p* < .001, *SRMR* = .05, *RMSEA* = .07, *NFI* = .94, C*FI* = .96, *GFI* = .96. All unstandardized parameter estimates were significant at *p* < .001 (see Figure 1 for the path diagram). We also compared this two‐factor model to a model in which the collective victim beliefs and collective resilience beliefs loaded onto one factor, *χ* ^2^ (16) = 96.43, *p* < .001, *SRMR* = .38, *RMSEA* = .12, *NFI* = .87, C*FI* = .90, *GFI* = .92. A Chi‐Square difference test between these models showed that the two‐factor solution was the better fitting model, Δχ^2*^ (1) = 51.22, *p* < .01.

The means, standard deviations and bivariate correlations of all key measures are displayed in Table 3. As seen in Table 4, collective victimhood and collective resilience beliefs were positively correlated. Next, we aimed to examine whether collective resilience beliefs uniquely predicted our outcomes of interest.

**TABLE 3:** Means, standard deviations and bivariate correlations between measured variables in Study 1

	*M* (*SD*)	1	2	3	4
1. Historical collective victimhood	6.35 (.84)	1			
2. Historical collective resilience	6.28 (.80)	.56***	1		
3. Collective continuity	5.23 (.99)	.31***	.37***	1	
4. Support for the Black Lives Matter movement	4.76 (1.83)	.21***	.17**	.29***	1

*Note*: The scale anchors were from 1 to 7.

**p < .05, ***p < .001.

**TABLE 4:** Means, standard deviations and bivariate correlations between measured variables in Study 2

	*M* (*SD*)	1	2	3	4
1. Historical collective victimhood	6.35 (.76)	1			
2. Historical collective resilience	6.38 (.74)	.50***	1		
3. Collective continuity	5.48 (.92)	.34***	.35***	1	
4. Support for the Black Lives Matter movement	5.35 (1.58)	.33***	.32***	.39***	1

*Note*: The scale anchors were from 1 to 7.

***p < .001.

#### Multiple regression analyses

First, to test the link between historical collective victimhood/historical collective resilience and each outcome of interest, we conducted a series of multiple linear regression analysis using the GLM procedure in SAS 9.4. The goal was to test our hypothesis that historical collective resilience would predict outcomes above and beyond the effect of historical collective victimhood. All analyses controlled for demographic variables (gender, age, income, political orientation). In line with expectations, we found that historical collective victimhood did not significantly predict collective continuity (*b* = .15, *SE* = .08, *t* = 1.77, *p* = .077, *η* ^*2*^ = .01); however, historical collective resilience predicted higher perceived collective continuity (*b* = .36, *SE* = .09, *t* = 4.19, *p* < .001, *η* ^*2*^ = .06). Surprisingly, neither historical collective victimhood (*b* = .29, *SE* = .16, *t* = 1.86, *p* = .064, *η* ^*2*^ = .01) nor historical collective resilience significantly predicted support for the Black Lives Matter movement (*b* = .16, *SE* = .16, *t* = .96, *p* = .337, *η* ^*2*^ = .004). Although there was an absence of a significant link between the predictor and outcome variable, we still tested for the presence of an indirect effect because we had an a priori mediation hypothesis (Agler & De Boeck, 2017). At times, indirect effects may be present even if there are no direct effects due to suppression effects (Hayes, 2017). However, given that we do not expect suppression effects, it is important to note that readers should be cautious in interpreting the indirect effect (see Shrout & Bolger, 2002).

#### Indirect effect analyses

**FIGURE 2:**
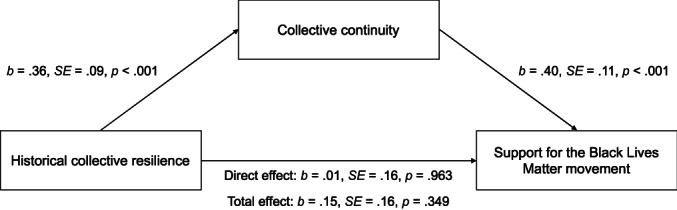
Mediation model for Study 1 showing historical collective resilience predicting support for the Black lives matter movement, via greater collective continuity. Historical collective victimhood, gender, age, income, and political orientation were entered as statistical controls

Next, to test our hypothesis that historical collective resilience beliefs (X) predicted support for BLM (Y) via collective continuity (M), we conducted indirect effects analyses using Model 4 of PROCESS 3.5 with 10,000 bootstrap samples (Hayes, 2017). Historical collective victimhood beliefs and demographic variables (gender, age, income, political orientation) were entered as covariates. Figure 2 shows the results of the mediation analysis. There was a significant indirect effect of historical collective resilience on support for the Black Lives Matter movement via collective continuity, *IE* = .14, boot *SE* = .06, boot 95% *CI* [.0469, .2976].

### Discussion

The qualitative results from Study 1 showed that historical collective victimhood and historical collective resilience were both present in Black Americans' collective memories of their history. The various themes we identified showed that victimization and resilience can surface in different ways. Notably, it is not merely the explicit suffering that constitutes victimization, but also symbolic exclusion and the ways in which discrimination is institutionalized. As for resilience, it was not only about the collective resistance efforts of Black people in the past, but also commemorating the accomplishments of Black people including notable figures who were paved the way for others. These findings point to potential gaps in the ways in which we conceptualize resilience (Denham, 2008; Ramirez & Hammack, 2014; Wexler, 2014), as it is not only a matter of surviving and coping with oppression, but also thriving and celebrating the success of one's group within this context. In addition, there were also interconnections between collective victimhood and resilience such that resilience was often framed as a response to victimhood—which is consistent with prior research on the social representations of history amongst oppressed groups (e.g. Brasil & Cabecinhas, 2017; Neufeld & Schmitt, 2019).

In line with the qualitative findings and expectations, quantitative analyses also showed that a sense of historical collective victimhood and historical collective resilience were related but distinct constructs. Crucially, both historical narratives were differentially related to key outcomes relevant to current responses to injustice. Specifically, supporting our hypotheses, historical collective resilience (but not victimhood) predicted higher levels of collective continuity, which, in turn, predicted greater support for the ongoing Black Lives Matter movement. These quantitative findings showed that historical collective resilience in indirectly linked to the current struggle for social change in the Black community, in part due to the connection that participants perceive between the group's past and present. This finding is in line with recent work suggesting the importance of historical narratives to collective action (Freel & Bilali, 2021).

However, the link between historical collective resilience and support for the movement is unclear. Although at the level of bivariate correlations, there was a small positive correlation between historical collective resilience and support for the Black Lives Matter movement, in our mediation analysis, contrary to hypothesis, there was no significant direct effect between the two. This makes it important to replicate the indirect effect we found. Furthermore, we found relatively modest interrater reliability for the qualitative analyses, which may be because we were in the process of developing and refining these codes for the first time.

Building on Study 1, the goal of Study 2 was to replicate these findings in a new sample of Black Americans. We sought to examine more closely how Black Americans viewed the time period of the Civil Rights era. We selected this period in history because (1) it represents a time of significant struggle but also progress for the Black community and (2) this era was mentioned as a major topic in Study 1 when participants were asked to freely describe important moments in Black history. Specifically, we first examined the extent to which participants freely recalled events that can be construed as victimization and/or resilience. Next, we also sought to replicate the positive links between historical collective resilience (versus victimhood) on support for the Black Lives Matter movement via greater collective continuity.

## STUDY 2

### Method

#### Participants

A total of 303 Black American participants were recruited from the Cloud Research platform. As a selection criterion on the recruitment platform, we specified that participants who took part in Study 1 would not be allowed to participate in Study 2. Again, sample size was determined based on cost and convenience. However, 9 participants did not respond to the writing prompt and were, therefore, excluded from analyses. Thus, 294 participants were retained for analyses. Participants' age ranged between 19 to 74 years (*M*_age_ = 36.25, *SD*_age_ = 11.20). There were 208 females, 85 males and one identified as non‐binary/third gender. Participants' median annual household income was between $40,000 to $59,999 with income brackets ranging between less than $19,999 to over $100,000.

#### Power analysis

A sensitivity analysis with G*Power (Faul et al., 2007) indicated that with a sample size of 294, we would have been able to detect a bivariate correlation of at least .16 with a two‐tailed α of .05 at 80% power, which is below the smallest correlation of interest that we found (*r* = .32 between historical collective resilience and support for the Black Lives Matter movement), suggesting that the study was sufficiently powered.

#### Procedure

We followed a similar procedure to Study 1. The only difference was that the open‐ended question was changed to be specifically about the events from the Civil Rights era, instead of generally asking participants to discuss any aspect of Black history as we did in Study 1. Participants were asked to ‘list three specific events that come to mind from the Civil Rights era in the 1950s and ‘60s’. Participants were instructed to write one event per line, and that the events did not need to be in chronological order. After the writing prompt, participants responded to the same measures asked in Study 1: collective victimhood beliefs (α = .62), collective resilience beliefs (α = .73), collective continuity (α = .84), support for the Black Lives Matter movement (α = .88) and a series of demographic questions.

### Results

#### Qualitative findings: Representations of the Civil Rights era

##### Analytical approach

All open‐ended responses were coded through content analysis using theoretically driven codes developed based on Study 1.^3^ The collective victimhood theme referred to events that mentioned discrimination, loss and suffering; the collective resilience theme included events that were about Black leadership, social movements and collective action.

Since participants were asked to list three events, each participant received three codes, one for each event (1 = theme was mentioned, 0 = theme was not mentioned). There were 17 responses that were not codable because it was ambiguous and did not refer to a specific event (e.g. ‘war’, ‘lawsuit’). There were also 11 responses that were left blank (i.e. there were 10 participants who listed fewer than three events). The responses were independently coded by the third author and a research assistant. There was a high level of agreement (see Altman, 1990; Fleiss & Cohen, 1973; Landis & Koch, 1977) between coders for the first code, κ = .84 (95% *CI*, .7804 to .9091), *p* < .001, the second code, κ = .87 (95% *CI*, .8063 to .9253), *p* < .001 and the third code, κ = .93 (95% *CI*, .8940 to .9737), *p* < .001. Differences were resolved through discussion. Next, we further condensed the codes such that each participant received a code for each theme based on whether the theme was mentioned at least once.

##### Findings

First, in total 62.24% of participants mentioned themes of collective victimhood (*n* = 183). These events included the murder of Emmett Till, the lynching of Blacks, the Birmingham church bombings by White supremacists, police brutality against peaceful protests (e.g. Bloody Sunday), the assassinations of Martin Luther King Jr. and Malcom X, Jim Crow laws and various segregation practices in public life. These topics were consistent with the subthemes mentioned in Study 1 under the theme of historical collective victimhood.

Second, there were 92.52% that mentioned themes of collective resilience (*n* = 272). These events referred to the actions of prominent Black leaders (e.g. Martin Luther King, Rosa Parks, Malcom X), collective action (e.g. the March on Selma, the March on Washington, sit‐ins, bus boycotts, the freedom rides, the Little Rock Nine school desegregation efforts) and social movements (e.g. the Black Panther movement, the Chicago Freedom movement, the Civil Rights movement). Once again, these topics were consistent with the subthemes mentioned in Study 1 under the theme of historical collective resilience. Further, it is worth noting that 54.76% of participants (*n* = 161) mentioned both themes of collective victimhood and resilience. This is in line with the findings from Study 1, which showed a significant overlap in participants who mentioned both historical narratives.

#### Quantitative findings: The link between historical collective resilience and present‐day responses to injustice

**FIGURE 3:**
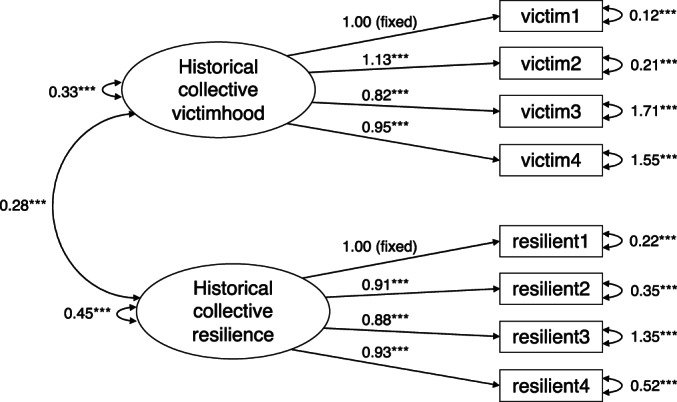
Two‐factor confirmatory factor analysis of historical collective victimhood and historical collective resilience for Study 2

As in Study 1, we conducted a CFA of a two‐factor solution with the collective victim belief items and the collective resilience belief items loading onto two separate latent factors. We fixed the loading of one of the indicators for each factor to a constant (i.e. 1) (see Kline, 2015). The model fit was acceptable, *χ* ^2^ (17) = 81.11, *p* < .001, *SRMR* = .06, *RMSEA* = .11, *NFI* = .89, C*FI* = .92, *GFI* = .93. All unstandardized parameter estimates were significant at *p* < .001 (see Figure 3 for the path diagram). As we did in Study 1, we also compared this two‐factor model to a model in which the collective victim beliefs and collective resilience beliefs loaded onto one factor, *χ* ^2^ (16) = 189.04, *p* < .001, *SRMR* = .66, *RMSEA* = .17, *NFI* = .76, C*FI* = .77, *GFI* = .88. A Chi‐Square difference test between these models showed that the two‐factor solution was the better fitting model, Δχ^2*^ (1) = 107.93, *p* < .01.

The means, standard deviations and bivariate correlations of key measures are displayed in Table 4. As seen in Table 4, historical collective victimhood and historical collective resilience were positively correlated. These descriptive findings are in line with those of Study 1.

##### Multiple regression analyses

As we did in Study 1, we tested the link between historical collective victimhood/historical collective resilience and each outcome of interest by conducting a series of multiple linear regression analysis using the GLM procedure in SAS 9.4. All analyses controlled for demographic variables (gender, age, income, political orientation). We found that historical collective victimhood predicted greater perceived collective continuity (*b* = .25, *SE* = .08, *t* = 3.37, *p* < .001, *η* ^*2*^ = .04), and historical collective resilience also predicted higher collective continuity (*b* = .31, *SE* = .08, *t* = 4.03, *p* < .001, *η* ^*2*^ = .05). Both historical collective victimhood (*b* = .48, *SE* = .13, *t* = 3.69, *p* < .001, *η* ^*2*^ = .05) and historical collective resilience predicted greater support for the Black Lives Matter movement (*b* = .43, *SE* = .13, *t* = 3.23, *p* = .001, *η* ^*2*^ = .04).

##### Indirect effect analyses

**FIGURE 4:**
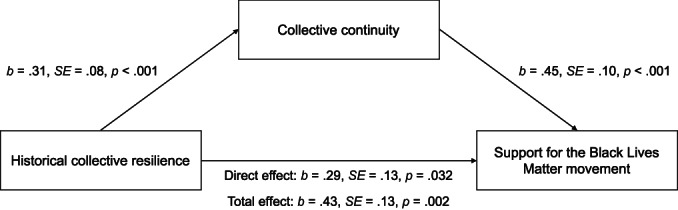
Mediation model for Study 2 showing historical collective resilience predicting support for the Black lives matter movement, via greater collective continuity. Historical collective victimhood, gender, age, income, and political orientation were entered as statistical controls

Next, as we did in Study 1, to test whether historical collective resilience beliefs (X) predicted support for BLM (Y) via collective continuity (M), we conducted an indirect effects analysis using Model 4 of PROCESS 3.5 with 10,000 bootstrap samples (Hayes, 2017). Historical collective victimhood and demographic variables (gender, age, income, political orientation) were entered as covariates. Figure 4 shows the results of the mediation analysis. There was a significant indirect effect of historical collective resilience on support for the Black Lives Matter movement via collective continuity, *IE* = .14, boot *SE* = .05, boot 95% *CI* [.0625, .2576].

### Discussion

In line with Study 1 and as hypothesized, Study 2 found that although collective victimhood was an important part of people's collective memory of the Civil Rights era, collective resilience was also an important part of it. Specifically, qualitative analyses revealed that participants readily recalled both events of collective victimization and resilience in their group's history. There was also a high level of co‐occurrence such that many participants who listed events illustrative of collective victimhood also listed events reflecting collective resilience. This further corroborates the findings from Study 1, which showed that collective victimhood and resilience were interconnected narratives of Black history. These findings are consistent with past research demonstrating how narratives of resilience are evoked when discussing collective victimization experiences (Vollhardt & Nair, 2018) as well as a group's historical trauma (Hatala et al., 2016; Ramirez & Hammack, 2014; Wexler, 2014).

In addition, the quantitative results from Study 2 largely replicated the findings of Study 1. Historical collective resilience predicted higher levels of collective continuity, which, in turn, predicted greater support for the ongoing Black Lives Matter movement. However, it is important to note that unlike Study 1, in Study 2 we did not counterbalance the order in which participants were asked to reflect on their collective memories. Participants were always led to think about their group's history at the beginning of the survey. It is, therefore, possible that we have inadvertently primed participants to think about how past experiences were linked to present‐day struggles. This difference in the survey flow for both studies may explain why we found a significant link between historical collective resilience and support for the Black Lives Matter movement for Study 2 but not in Study 1. Furthermore, Study 2 focused specifically on the Civil Rights era, which may have prompted participants to think more about the themes of resilience rather than victimization. However, we note that it was also a time of intense opposition from White Americans during which Black people were treated with prejudice and hostility, including racial segregation, lynching and mob violence (e.g., Petersen & Ward, 2015).

One inconsistency between the findings in both studies is that unlike Study 1, Study 2 showed that historical collective victimhood also predicted higher levels of collective continuity. However, it is important to note that our findings controlled for the effects of historical collective victimhood, thereby showing the unique role of historical collective resilience in explaining greater support for the current struggle for social change in the Black community as hypothesized.

## GENERAL DISCUSSION

Groups that have long faced oppression often also have a history of being resilient, in terms of showing strength and empowerment in the face of injustice. In the present research, we examined the role of collective historical resilience, that is, the perception that one's group has a tradition of overcoming suffering and resisting oppression. Focusing on the context of Black Americans, as hypothesized, we found in both studies that when Black Americans were asked about their group's history, in addition to recalling instances of collective victimhood, they spontaneously recalled instances of collective resilience. This showed that collective historical resilience was a central part of the group's collective memory and co‐occurs with collective historical victimhood.

In line with hypotheses, we also found in both studies that beyond any effect of collective historical victimhood, perceiving collective historical resilience was linked to a greater sense of collective continuity (i.e. that the past, present and future of the group was interconnected), which, in turn, was linked to greater support for the ongoing Black Lives Matter movement. This finding underscored how remembering instances of past collective resilience gives rise to perceptions of continuity between past and present. In turn, this enhanced sense of collective continuity of the in‐group's history of resilience helps explain how present‐day group members mobilize for social change. In this way, collective resilience of the past continues to echo in resistance efforts of the present.

### Theoretical contributions and implications

Our findings make a number of important theoretical contributions. First, extant research has largely examined the negative impact of collective victimhood on intergroup relations (e.g., Bar‐Tal et al., 2009; Noor et al., 2012; Schori‐Eyal et al., 2014) and has tended to overlook the role of collective resilience (see Vollhardt & Nair, 2018 for a similar argument). These literatures have also underestimated the ability of oppressed groups to effectively cope with adversity (Leach & Livingstone, 2015). We complement this literature by demonstrating that oppressed groups also have a history of collective resilience as a key part of their collective memories, which can continue to inform present‐day attitudes and experiences.

Our findings are consistent with past research on how social representations of resilience are often present and linked to experiences of victimization in oppressed group's recollections of their history (e.g., Denham, 2008; Hatala et al., 2016; Muldoon et al., 2020; Ramirez & Hammack, 2014; Vollhardt & Nair, 2018). Our qualitative findings also contribute to prior theorizing on the content and function of collective resilience as including multi‐faceted components such as resistance/collective action, thriving, and collective autonomy, as well as celebrating both in‐group accomplishments and structural changes as collective victories.

In addition, our findings further establish a link between collective memories and a group's current responses to oppression. Most prior literature on social change processes has largely overlooked the critical role that perceptions of a group's history play in social mobilization (c.f., Cheung et al., 2017; Salter & Adams, 2016). Instead, collective action is often examined in relation to a single point in time, whereby only present‐day group perceptions and beliefs are considered. However, it is clear that a group's history offers a blueprint for how to deal with and respond to ongoing challenges (e.g., Liu & Hilton, 2005; de Saint‐Laurent & Obradović, 2019). The present research underscores the importance of considering groups as situated in their social and political history.

### Limitations and future directions

Although our research used a multi‐method approach, which provides greater confidence in our findings, our studies were cross‐sectional and correlational in design. We, therefore, cannot make causal claims. It is possible that supporting collective action for social change leads group members to re‐interpret the past and, therefore, construct narratives of collective resilience. Since collective memories can be strategically deployed and socially constructed to serve particular political purposes (e.g., Mols & Jetten, 2014; Obradović & Howarth, 2018), the iterative and dynamic nature of collective memories of resilience needs further research attention. It is also possible that the rise in the movement drives collective remembering of past events, which would suggest that support for the Black Lives Matter movement promotes a sense of collective continuity, which, in turn, raises collective historical resilience. This is consistent with the social movement literature that has shown how certain events in a group's collective memory can be used to mobilize people in the present (Gongaware, 2011; Harris, 2006). Beyond collective action outcomes, future research could investigate the health and well‐being benefits of collective historical resilience for present‐day group members (see Chandler & Lalonde, 1998; Wexler, 2014).

In addition, there are also methodological limitations in our studies. Across both studies, we found that participants reported very high levels of collective resilience and collective victimhood, which can create incorrect estimates in linear regressions. Future research could potentially change the anchors of the scales used or rephrase the way the questions are asked to capture the salience of these perceptions rather than level of agreement with each statement. Our linear regression approach also partials out the variance between collective resilience and collective victimhood, but arguably the overlap between the two is theoretically meaningful and important to explore (c.f., Vollhardt & Nair, 2018).

Our focus on the experiences of Black Americans has strong external validity and applied relevance to understanding the current Black Lives Matter movement, but our findings do not necessarily apply to other oppressed groups. It is possible that the sense of collective historical resilience may be found in groups that have similarly faced oppression throughout history, for example, Indigenous Australians who have a legacy of resisting British colonialism. In addition, when examining a collective historical resilience, it is also important not to overlook forms of everyday resistance that are hidden or disguised (Vollhardt et al., 2020), which we did not explicitly account for in the present research. For example, refusing to comply with authority or feigning ignorance to subvert unjust power (Rosales & Langhout, 2020) are instances of everyday resistance that people may have practiced historically, but it was not explicitly named or conceptualized as resistance. More generally, we need greater understanding of the form and function of collective historical resilience.

### Concluding remarks

To conclude, our research highlights the importance of considering narratives of resilience in a group's collective memory. Collective historical resilience is linked to a sense of connection between the past, present and future of a group, as well as subsequently shaping adaptive and resilient group‐based responses to ongoing oppression. Importantly, our findings point to the benefit of moving beyond studying oppressed groups through a myopic lens of victimhood alone, and instead simultaneously considering their collective strength and survival throughout history.

## AUTHOR CONTRIBUTIONS

**Hema Preya Selvanathan:** Conceptualization; data curation; formal analysis; investigation; methodology; writing – original draft; writing – review and editing. **Jolanda Jetten:** Conceptualization; funding acquisition; methodology; supervision; writing – review and editing. **Alexis Umeh:** Data curation; formal analysis; writing – review and editing.

## CONFLICT OF INTEREST

The authors declare that there are no conflicts of interest. This research was approved by the ethics committee in The University of Queensland, Australia.

## Data Availability

The survey material, data files, and analysis scripts are available on the Open Science Framework: https://osf.io/rhkpz/.

## Footnotes

^1^
It is possible that responses to the close‐ended measures may be influenced by the order in which participants completed the open‐ended and close‐ended measures. We, therefore, tested for any potential influence of order. We found no effect of order on levels of historical collective victimhood, historical collective resilience, and collective continuity. There was also no interaction between resilience and order in predicting collective continuity nor in predicting support for the Black Lives Matter movement. Further, participants were no more likely to report themes of victimization or resilience as a function of the order in which they responded to the open‐ended prompt.

^2^
During the initial coding process, we coded several subthemes that are now included in collective resilience as a separate theme on ‘collective victory’, which were elements of Black history that were celebrated and remembered as triumphant. We initially believed this to be somewhat distinct from collective resilience because the focus was on positive events alone typically by recounting ingroup success and milestones. However, upon further consideration during the review process, we believe that the collective victory subthemes should be part of the theme on collective resilience.

^3^
As in Study 1, we also coded participants' responses for collective victory, which was initially treated as a separate theme, but upon further consideration during the review process, we decided to combine these codes within the theme of collective resilience.
